# Supplementary material for: Anticoagulants as Potential SARS-CoV-2 Mpro Inhibitors for COVID-19 Patients: In Vitro, Molecular Docking, Molecular Dynamics, DFT, and SAR Studies
Source: Int J Mol Sci. 2022 Oct 13;23(20):12235. doi: 10.3390/ijms232012235 (PMC9603561; doi:10.3390/ijms232012235)
Supplement: Supplementary file 1 [file ijms-23-12235-s001.zip › ijms-1891072-supplementary.pdf]

Anticoagulants as Potential SARS-CoV-2 M^{pro} Inhibitors for COVID-19 Patients: In Vitro, Molecular Docking, Molecular Dynamics, DFT, and SAR Studies

Ayman Abo Elmaaty [1], Wagdy M. Eldehna [2,3], Muhammad Khattab [4], Omnia Kutkat [5], Radwan Alnajjar [6,7,8], Ahmed N. El-Taweel [5], Sara T. Al-Rashood [9], Mohammed A. S. Abourehab [10], Faizah A. Binjubair [9], Mohamed A. Saleh [11,12], Amany Belal [13,14] and Ahmed A. Al-Karmalawy [15,*]

[1] Department of Medicinal Chemistry, Faculty of Pharmacy, Port Said University, Port Said 42526, Egypt
[2] Department of Pharmaceutical Chemistry, Faculty of Pharmacy, Kafrelsheikh University, Kafrelsheikh 33516, Egypt
[3] School of Biotechnology, Badr University in Cairo, Badr City 11829, Egypt
[4] Department of Chemistry of Natural and Microbial Products, Pharmaceutical and Drug Industries Research Institute, National Research Centre, El-Buhouth St., Dokki, Cairo 12622, Egypt
[5] Center of Scientific Excellence for Influenza Viruses, National Research Centre, Giza 12622, Egypt
[6] Department of Chemistry, Faculty of Science, University of Benghazi, Benghazi 16063, Libya
[7] PharmD, Faculty of Pharmacy, Libyan International Medical University, Benghazi 16063, Libya
[8] Department of Chemistry, University of Cape Town, Rondebosch 7701, South Africa
[9] Department of Pharmaceutical Chemistry, College of Pharmacy, King Saud University, Riyadh 11451, United Arab Emirates
[10] Department of Pharmaceutics, Faculty of Pharmacy, Umm Al-Qura University, Makkah 21955, United Arab Emirates
[11] Department of Clinical Sciences, College of Medicine, University of Sharjah, Sharjah 27272, United Arab Emirates
[12] Department of Pharmacology and Toxicology, Faculty of Pharmacy, Mansoura University, Mansoura 35516, Egypt
[13] Medicinal Chemistry Department, Faculty of Pharmacy, Beni-Suef University, Beni-Suef 62514, Egypt
[14] Department of Pharmaceutical Chemistry, College of Pharmacy, Taif University, P.O. Box 11099, Taif 21944, Saudi Arabia
[15] Pharmaceutical Chemistry Department, Faculty of Pharmacy, Ahram Canadian University, 6th of October City, Giza 12566, Egypt
* Correspondence: akarmalawy@acu.edu.eg

Supplementary Data

Materials and Methods

SI 1: Molecular dynamics simulations

The MD simulations were carried out using Desmond simulation package of Schrödinger LLC [82]. The NPT ensemble with the temperature 300 K and a pressure 1 bar was applied in all runs. The simulation length was 200 ns with a relaxation time 1 ps for the ligands. The OPLS3 force field parameters were used in all simulations [90]. The cutoff radius in Coulomb interactions was 9.0 Å. The orthorhombic periodic box boundaries were set 10 Å away from the protein atoms. The water molecules were explicitly described using the transferable intermolecular potential with three points (TIP3P) model [91,92]. Salt concentration set to 0.15 M NaCl and was built using the System Builder utility of Desmond [93]. The Martyna−Tuckerman−Klein chain coupling scheme with a coupling constant of 2.0 ps was used for the pressure control and the Nosé−Hoover chain coupling scheme for the temperature control [94,95]. Nonbonded forces were calculated using a RESPA integrator where the short-range forces were updated every step and the long-range forces were updated every three steps. The trajectories were saved at 20 ns intervals for analysis. The behavior and interactions between the ligands and protein were analyzed using the Simulation Interaction Diagram tool implemented in Desmond MD package. The stability of MD simulations was monitored by looking on the RMSD of the ligand and protein atom positions in time.

SI 2: MD trajectory analysis and prime MM-GBSA calculations

Simulation interactions diagram panel of Maestro software was used to monitoring interactions contribution in the ligand-protein stability. The molecular mechanics generalized born/solvent accessibility (MM – GBSA) was performed to calculate the ligand binding free energies and ligand strain energies for docked compounds over the last 50 ns with thermal_mmgbsa.py python script provided by Schrodinger which takes a Desmond trajectory file, splits it into individual snapshots, runs the MM-GBSA calculations on each frame, and outputs the average computed binding energy.

SI 3: SARS-CoV-2 M^{pro} inhibitory assay

The *3CL Protease Assay Kit* is designed to measure 3CL Protease activity for screening and profiling applications, in a homogeneous assay with no time-consuming washing steps. The kit comes in a convenient 96-well format, with purified 3CL Protease, fluorogenic substrate, and

3CL Protease assay buffer for 100 enzyme reactions. 3CL inhibitor GC376 is also included as a positive control.

Protocol

Add **0.5 M DTT** to **3CL Protease Assay Buffer** so final DTT concentration is 1 mM. For example, add 10 µl of **0.5 M DTT** to 5 ml assay buffer. (DTT should be added just before use. Prepare only enough DTT-containing buffer as required for the assay. Store the remaining assay buffer at -20°C).

2) Thaw **3CL Protease** on ice. Upon first thaw, briefly spin tube containing enzyme to recover the full content of the tube. Aliquot **3CL Protease** into single use aliquots. Store remaining undiluted enzyme in aliquots at -80°C. Note: **3CL Protease** enzyme is sensitive to freeze/thaw cycles. Do not re-use dilute enzyme.
3) Dilute **3CL Protease** in **Assay buffer** (with 1 mM DTT) at 3-5 ng/µl (90-150 ng per reaction).

4) Add 30 µl **diluted 3CL Protease** enzyme solution to wells designated as "Positive Control", "Inhibitor Control" and "Test Sample". Add 30 µl **Assay buffer** (with 1 mM DTT) to the "Blank" wells.

**Component**	**Positive Control**	**Test Sample**	**Inhibitor Control**	**Blank**
3CL Protease (3-5 ng/µl)	30 µl	30 µl	30 µl	–
Assay Buffer (with DTT)	–	–	–	30 µl
GC376 (500 µM)	–	–	10 µl	–
Test Inhibitor	–	10 µl	–	–
Inhibitor Buffer (no inhibitor)	10 µl	–	–	10 µl
Substrate solution	10 µl	10 µl	10 µl	10 µl
**Total**	**50 µl**		**50 µl**	**50 µl**

5) Dilute 50 µg **GC376** in 200 µl water to obtain a 500 µM solution. Aliquot and store remaining solution in aliquots at -80°C. Add 10 µl **GC376** (500 µM) to the wells labeled "Inhibitor Control".
6) Prepare the inhibitor solution.

The final concentration of DMSO in the assay should not exceed 1%. If the inhibitor compound is dissolved in DMSO, make a 100-fold higher concentration of the compound than the highest concentration you want to test in DMSO. Then make a 20-fold dilution in 1X assay buffer (at this step the compound concentration is 5-fold higher than the final concentration).

If the inhibitor compound is dissolved in water, make a solution of the compound 5-fold higher than the final concentration in 3CL Protease assay buffer (with 1 mM DTT). For example, diluting 50 μg GC376 in 200 μl water (step 5) creates a 500 μM solution. Adding 10 ul to the assay (final volume 50 μl) results in a 100 μM final concentration.

7) Add 10 μl inhibitor to each well designated "Test Sample". Add 10 μl 1X assay buffer or 5% DMSO (depending on which inhibitor solution is used) to "Blank" and "Positive Control" wells.
8) Preincubate enzyme with the inhibitor for 30 min at room temperature with slow shaking.

9) Dilute 5 mM **3CL Protease substrate** 1:20 in assay buffer with DTT, to make a 250 μM solution. Dilute only enough as is required for the assay.
10) Start reaction by adding 10 μl of the substrate solution to each well (Final concentration of the **3CL Protease substrate** in a 50 μl reaction is 50 μM).
11) Incubate at room temperature for overnight. Seal the plate with the plate sealer. Measure the fluorescence intensity in a microtiter plate-reading fluorimeter capable of excitation at a wavelength 360 nm and detection of emission at a wavelength 460 nm. The fluorescence intensity can also be measured kinetically. "Blank" value is subtracted from all other values.

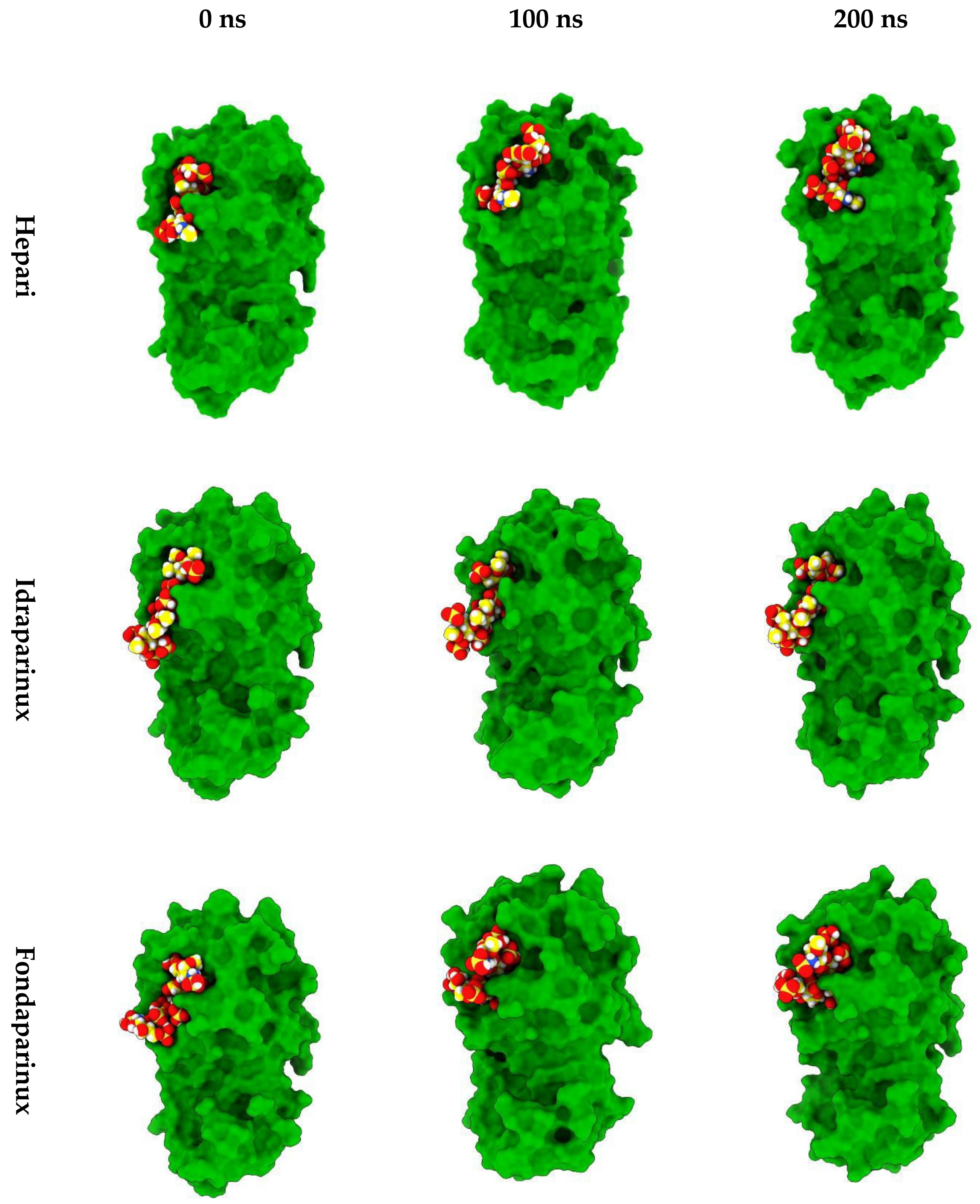

Figure SI 1. Ligand position inside the active site at 0, 100, and 200 ns.

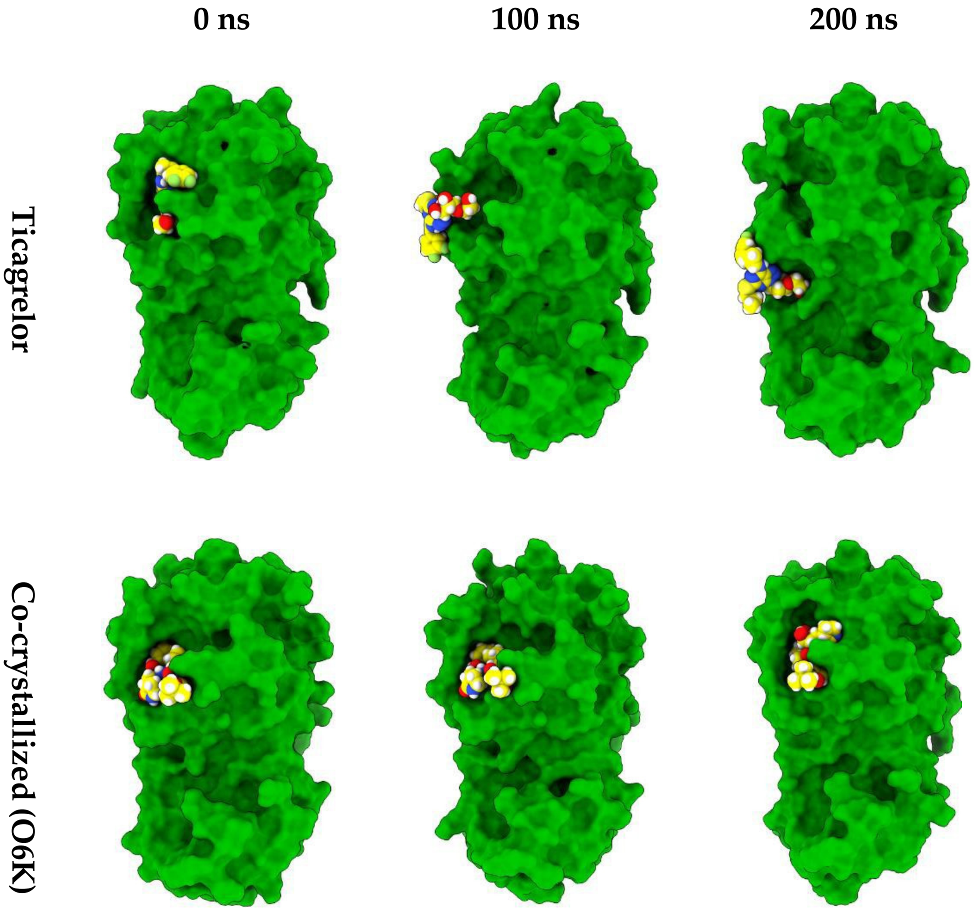

Figure SI 1 Continue. Ligand position inside the active site at 0, 100, and 200 ns.

References

90. Harder, E.; Damm, W.; Maple, J.; Wu, C.; Reboul, M.; Xiang, J.Y.; Wang, L.; Lupyan, D.; Dahlgren, M.K.; Knight, J.L.; et al. OPLS3: A Force Field Providing Broad Coverage of Drug-like Small Molecules and Proteins. *J. Chem. Theory Comput.* **2016**, *12*, 281–296, https://doi.org/10.1021/acs.jctc.5b00864.
91. Jorgensen, W.L.; Chandrasekhar, J.; Madura, J.D.; Impey, R.W.; Klein, M.L. Comparison of simple potential functions for simulating liquid water. *J. Chem. Phys.* **1983**, *79*, 926–935. https://doi.org/10.1063/1.445869.
92. Neria, E.; Fischer, S.; Karplus, M. Simulation of activation free energies in molecular systems. *J. Chem. Phys.* **1996**, *105*, 1902–1921, https://doi.org/10.1063/1.472061.
93. Manual, D.U. Desmond2. 2. 2009.
94. Martyna, G.J.; Klein, M.L.; Tuckerman, M. Nosé–Hoover chains: The canonical ensemble via continuous dynamics. The Journal of chemical physics **1992**, *97*, 2635–2643.
95. Martyna, G.J.; Tobias, D.J.; Klein, M.L. Constant pressure molecular dynamics algorithms. *J. Chem. Phys.* **1994**, *101*, 4177–4189, https://doi.org/10.1063/1.467468.
